# Emerging Lipid Targets in Glioblastoma

**DOI:** 10.3390/cancers16020397

**Published:** 2024-01-17

**Authors:** Ammar Darwish, Milán Pammer, Ferenc Gallyas, László Vígh, Zsolt Balogi, Kata Juhász

**Affiliations:** 1 Institute of Biochemistry and Medical Chemistry, Medical School, University of Pécs, 7624 Pécs, Hungary; 2 Institute of Biochemistry, HUN-REN Biological Research Center, 6726 Szeged, Hungary

**Keywords:** GBM, tumor hallmarks, tumor lipid, lipid therapy

## Abstract

**Simple Summary:**

Glioblastoma (GBM) is a deadly cancer type of the brain with an average of 12 months of survival after diagnosis. Current clinical therapies generally provide only a short lifetime extension. GBM is embedded in a highly lipid-rich environment, and emerging evidence supports that lipid-based therapeutic molecules are promising research targets to unravel novel drugs. Recent efforts of phase trials suggest that lipid-based combination therapies may offer a survival benefit. Here we review preclinical and clinical antitumor approaches targeting the altered lipid metabolism of glioblastoma.

**Abstract:**

GBM accounts for most of the fatal brain cancer cases, making it one of the deadliest tumor types. GBM is characterized by severe progression and poor prognosis with a short survival upon conventional chemo- and radiotherapy. In order to improve therapeutic efficiency, considerable efforts have been made to target various features of GBM. One of the targetable features of GBM is the rewired lipid metabolism that contributes to the tumor’s aggressive growth and penetration into the surrounding brain tissue. Lipid reprogramming allows GBM to acquire survival, proliferation, and invasion benefits as well as supportive modulation of the tumor microenvironment. Several attempts have been made to find novel therapeutic approaches by exploiting the lipid metabolic reprogramming in GBM. In recent studies, various components of *de novo* lipogenesis, fatty acid oxidation, lipid uptake, and prostaglandin synthesis have been considered promising targets in GBM. Emerging data also suggest a significant role hence therapeutic potential of the endocannabinoid metabolic pathway in GBM. Here we review the lipid-related GBM characteristics in detail and highlight specific targets with their potential therapeutic use in novel antitumor approaches.

## 1. Introduction

As a result of the brain’s complex and fundamental role, any inconsistent growth or metabolic alterations may lead to severe consequences. Brain tumors are considered among the top ten deadliest cancers, despite accounting for only 2% of total cases. High mortality rates are generally attributed to late diagnosis and lower efficiency of current surgical, chemo-, and radiotherapeutic treatments [1,2]. Primary brain tumors originate from cells within the central nervous system, while secondary brain tumors originate from other primary cancers, most commonly from breast cancer, lung cancer, or melanoma [3,4]. Based on the rate of proliferation and probability of recurrence, there are benign tumors characterized by slower growth and better treatment outcome, and malignant tumors marked by poor prognosis due to their rapid proliferation and intensive local spreading, which rarely affect distant organs [5]. Malignant tumors of the brain include gliomas, lymphomas, and hematopoietic neoplasm. Gliomas are the most frequent malignant brain tumors as they account for approximately 80% of total cases [6]. This group of tumors arises from neuroglial stem or neuronal progenitor cells. Based on the cell of origin, oligodendrogliomas, ependymomas, and astrocytomas can be distinguished [7]. Gliomas can also be classified into four main groups according to the WHO 2021 Classification of Tumors of the Central Nervous System [8]. The most severe type of glioma is glioblastoma (GBM), which is known for its severity, poor prognosis, and limited therapeutic efficacy. Therefore, extensive attempts have been made to find alternative treatment protocols. Multiple omics studies have recently been performed to help understand the molecular changes and metabolic reprogramming of GBM. Besides proteins and nucleic acids, lipids have been raised to play a fundamental role in tumor pathogenesis. It is worth mentioning that antitumor effects of targeting different lipid metabolism pathways have been shown for several cancer types such as breast cancer, leukemia, prostate cancer, and GBM. In this review, we focus on the role of lipid metabolism and specific lipids related to glioblastoma.

## 2. Glioblastoma

**Figure 1:**
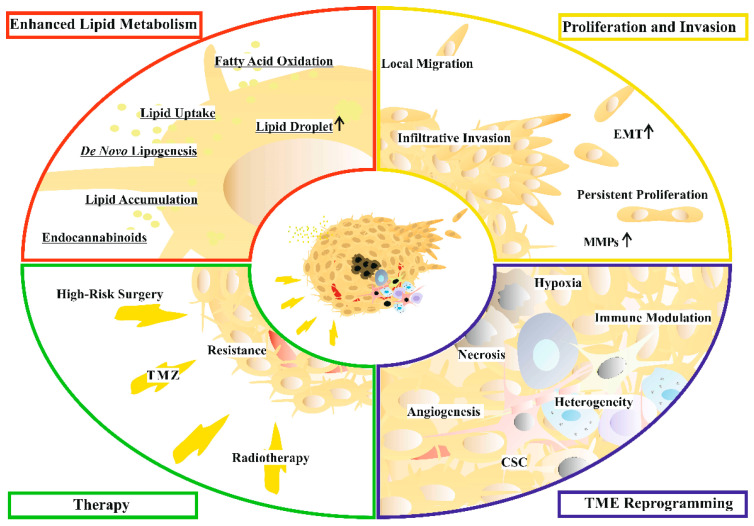
Targetable GBM hallmarks and features. Altered cellular processes related to cancer hallmarks of enhanced lipid metabolism (red), aggressive proliferative and invasive phenotype (yellow), reprogrammed tumor microenvironment (blue), and current therapy (green) of glioblastoma (GBM) are indicated in separate quarters. Features with typically elevated levels are marked with an arrow. Underlined features of the reprogrammed lipid metabolism are discussed in detail in the text. Abbreviations: CSC: cancer stem cell, EMT: epithelial–mesenchymal transition, MMPs: matrix metalloproteinases, TME: tumor microenvironment, and TMZ: temozolomide.

The majority of malignant brain tumors are GBM [9]. Several studies reported a prevalence ranging between 0.85 and 4.17 per 100,000 persons/year [10] with poor survival and prognosis as the main characteristics of GBM. A recent systematic review reported that 2-year, 3-year, and 5-year survival rates are 18%, 11% and 4%, respectively [11]. Meanwhile, the 10-year survival rate was shown to be 0.71% elsewhere [12]. A median estimated survival time of 14.6 months was found in patients who had been treated with a combination of radiotherapy and temozolomide (TMZ) [13]. Based on its severity, WHO classified GBM as a grade IV tumor [8]. The poor prognosis of GBM patients is explained by the high rate of tumor recurrence [14]. Despite the current clinical settings and substantial efforts, the vast majority of patients relapse, leading to a high mortality rate [15]. Many hallmarks of GBM contribute to poor prognosis, including therapy-induced resistance, modulation of the tumor microenvironment, invasion of neighboring tissues, and extensive metabolic reprogramming (Figure 1). 

Most GBM cases are primary neoplastic changes that usually affect elderly people [16]. A smaller number of GBM appear as secondary tumors originating from previously diagnosed low-grade (II or III) glioma. Although GBM is very aggressive and known for its ability to invade adjacent tissues, distant metastases (e.g., bone marrow or lung) are rarely formed [17]. A high degree of heterogeneity necessitated several classification systems for GBM. Based on their transcriptional features, GBM is divided into four categories: proneural, neural, classic, and mesenchymal. Among these classes, proneural GBM has the best prognosis with 17 months of median survival, while the mesenchymal form has the worst outcome [18,19]. Based on isocitrate dehydrogenase (IDH), GBM is classified into IDH-wild type (in which IDH is overexpressed) and IDH-mutant [20], which are associated with worse or better prognosis, respectively [21]. 

Glioma cells can penetrate the neighboring tissues through either vasculature beds or axons of the white matter [22]. Glioma cells can shrink and release free cytoplasmic water up to 33% of their volume and Cl^−^ ions through channels and transporters, allowing them to more easily cross physical barriers. Another mechanism involves the upregulation of matrix metalloproteinases that enable the efficient degradation of the extracellular matrix. Stiffness of the extracellular matrix is a key regulator of glioma invasion. Therefore, reduction in Cl^−^ levels or inhibition of matrix metalloproteinase-9 led to an anti-migratory effect on glioma cells [23,24,25]. GBM cells may also migrate via the epithelial–mesenchymal transition mechanism, supported by a very low expression of E-cadherin, functioning for cell–cell adhesion and contact prevention of migration [26]. It was also shown that epithelial–mesenchymal transition is activated in some GBM clones, which correlated with a poorer prognosis and a more aggressive tumor phenotype [27]. 

GBM is histologically diagnosed via biopsy either after the debulking surgery or via a stereotactic procedure, as well as some imaging techniques such as magnetic resonance imaging [7] or positron emission tomography [28]. Standard care comprises surgery, radiotherapy, and chemotherapy with TMZ [29]. Although removal of the entire tumor is impossible due to the infiltrative behavior of GBM [30], surgery aims to remove as much tumor tissue as possible without affecting healthy tissues. TMZ is a lipophilic DNA alkylating agent capable of crossing the blood–brain barrier and also improving the prognosis if combined with radiotherapy, and therefore has been widely used against GBM in clinical settings [13]. Unfortunately, half of the patients do not respond and develop resistance to TMZ [31]. To overcome the current therapeutic limitations, remarkable progress has been made in the last decade to explore the mechanisms of GBM pathogenesis. Several therapeutic opportunities have been investigated, such as immune therapies targeting epidermal growth factor receptors or vascular endothelial growth factor. In this respect, the humanized monoclonal antibody bevacizumab, an angiogenesis inhibitor, has been used as a first-line therapy for solid tumors characterized by poor survival. This type of treatment was approved by the Food and Drug Administration for metastatic colorectal and non-small-cell lung cancer as well as for recurrent GBM [32,33,34]. Attempts have also been made to treat GBM by exploiting the metabolic reprogramming reported for GBM. Targetable changes in lipids and their metabolites, which have been related to the pathogenesis and progress of GBM, are discussed below.

## 3. Lipids of the Healthy Brain

Lipid content in the brain is heterogeneous and accounts for 36–40%, 49–66%, and 8–81% of gray matter, white matter, and myelin, respectively [35]. The brain can synthesize fatty acids (FAs) such as monounsaturated (MUFAs) and saturated FAs by *de novo* synthesis, whereas polyunsaturated fatty acids (PUFAs) are taken from dietary resources only. Lipids of the brain consist of sphingolipids, glycerophospholipids (phospholipids), sterols, glycolipids, triacylglycerols, prenols, eicosanoids, and endocannabinoids (ECs) [36]. A large fraction of the brain lipids are plasmalogens that are composed of ether-linked fatty alcohols at the *sn-1* position [37]. Due to their prominent roles, cholesterol, phospholipids, and sphingolipids are considered as main lipids of the brain [38]. Brain cholesterol levels represent 23% of the total cholesterol content in the body [39]. Since the blood–brain barrier excludes cholesterol-carrying lipoproteins [40], cholesterol is synthesized by oligodendrocytes, astrocytes, and neurons [41]. Astrocytes also synthesize apolipoprotein E, which is responsible for the intercellular transportation of cholesterol [42].

Phospholipids include phosphatidylcholine, phosphatidylethanolamine, and phosphatidylserine, which are considered as main components of the cellular membranes, with a crucial role in membrane permeability barrier function and in regulating fluidity. Phospholipids account for as much as 25% of the dry weight of the brain [43]. They are cleaved by phospholipases such as phospholipase A2 (PLA_2_), releasing arachidonic acid (AA), which in turn is metabolized into eicosanoids that have different roles in the brain, mediating inflammation and sleep regulation [44,45]. Sphingolipids are characterized by a backbone of a sphingosine basis and include ceramide, sphingomyelin, ganglioside, and cerebroside. They are the primary component of the myelin membrane that insulates the axons [46]. Sphingolipids also coordinate cellular activities like differentiation and cell death in the central nervous system [47]. Brain lipids also include ECs, originally defined as endogenous lipid-based neurotransmitters involved in different physiological processes by mediating retrograde neurotransmission in a receptor-dependent manner [48,49]. Together with the endocannabinoid receptors and enzymes, they form the endocannabinoid system. Anandamide (AEA) and 2-arachidonoyl glycerol (2-AG) activate the cannabinoid receptors and their metabolism generates AA [50]. ECs coordinate different psychological functions such as hunger, emotional status, inflammation, and pain sensation [51]. The role and metabolism of ECs in the brain is one of the most investigated topics of neuroscience and has been summarized elsewhere [52].

## 4. Lipid Metabolism in Glioblastoma Cells

**Figure 2:**
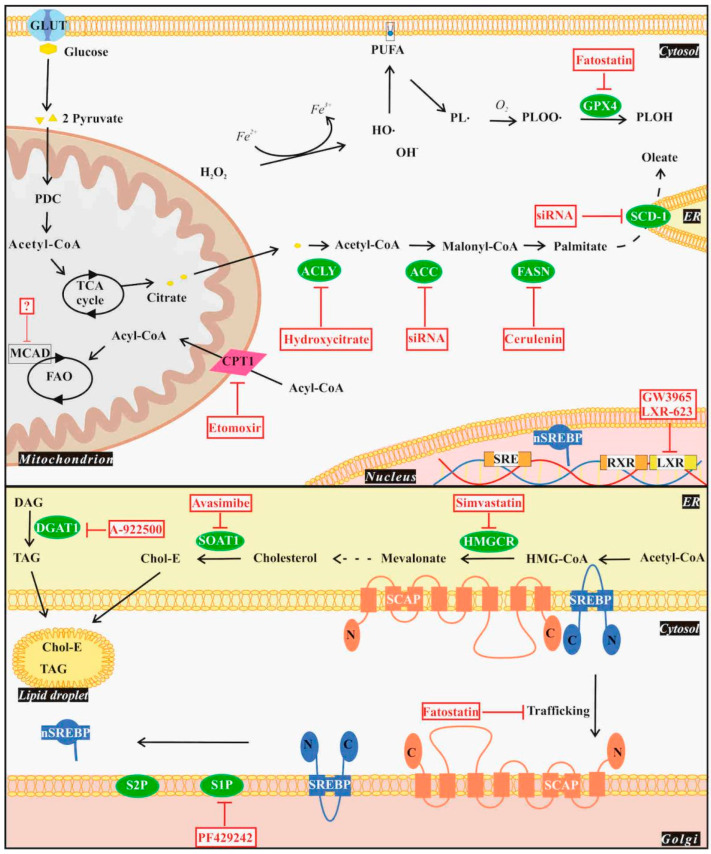
Experimental drug targets of the lipid metabolism pathways in GBM. Drugs affecting lipid metabolism pathways in GBM could target SREBP activation, *de novo* lipogenesis (DNL), lipid droplet (LD) formation, fatty acid oxidation (FAO), and ferroptosis. SREBP activation requires SREBP-SCAP translocation from ER to Golgi, where it is cleaved by S1P and S2P yielding the N terminal fragment of SREBP, which in turn translocates into the nucleus (nSREBP) and activates lipogenic genes. Fatostatin and PF429242 affect the SREBP pathway by inhibiting SREBP translocation and S1P activity, respectively. SREBP activation leads to DNL by using glycolysis-derived citrate as a precursor. Conversion of cytosolic citrate to fatty acids can be targeted by inhibiting the involved enzymes ACLY, ACC, or FASN by hydroxycitrate, siRNA, or cerulenin, respectively. Similarly, silencing expression of the ER-associated SCD-1 results in DNL inhibition. Simvastatin targets HMGCR and depletes cholesterol, which is crucial for GBM survival. To reduce lipotoxicity, GBM cells accumulate LDs and sequester excess FAs and lipids in the form of cholesteryl esters (Chol-E) or TAGs generated by the enzymes SOAT1 or DGAT1, respectively. LD formation can be targeted by inhibition of SOAT1 or DGAT1 by avasimibe or A-922500, respectively. Inhibition of the energy-producing FAO can be achieved by targeting CPT1 with etomoxir or by depleting MCAD, efficiently reducing GBM cell viability. Upon ferroptosis hydroxyl radicals react with PUFAs in the cell membrane, resulting in the generation of free cytotoxic radicals (PLOO·). GBM cells can escape ferroptosis through GPX4 activity by reducing PLOO·s to PLOH. This step can be blocked by the inhibitor fatostatin. Reducing LDLR expression through LXR elements by synthetic LXR agonists LXR-623 and GW3965 causes cholesterol-dependent GBM cell death. Abbreviations: ACC: acetyl-CoA carboxylase, ACLY: ATP citrate lyase, CPT1/2: carnitine palmitoyl transferase 1/2, DAG: diacylglycerol, DGAT: diacylglycerol acyltransferase, FASN: fatty acid synthase, GPX4: glutathione peroxidase 4, HMGCR: β-hydroxyl-β-methylglutaryl-CoA reductase, LDLR: low-density lipoprotein receptor, LXR: Liver X receptors, MCAD: medium-chain acyl-CoA dehydrogenase, PDC: pyruvate dehydrogenase complex, PL.: phospholipid radical, PLOH: phospholipid alcohol, PLOO.: phospholipid peroxyl radical, PUFA: polyunsaturated fatty acid, RXR: retinoid X receptor, S1P: site-1 protease, S2P: site-2 protease, SCAP: SREBP cleavage activating protein, SCD: stearoyl-CoA desaturase, SOAT: sterol O-acyltransferase, SREBP: sterol regulatory element binding protein, TAG: triacylglycerol, and TCA: tricarboxylic acid cycle.

Lipids account for 35% of the dry weight of GBM cells and have been recently revealed to exert an important role in GBM [53]. Glial cells are known for their ability to synthesize lipids such as FAs [54] and cholesterol [55] via *de novo* lipogenesis (DNL). Several studies showed that glioblastoma cells have irregular lipid metabolism to fulfill survival and progression requirements and to adapt to the tumor microenvironmental conditions, such as limited nutrients [56,57], making them independent of the exogenous lipid supply [58]. In this context, altered lipid metabolism in GBM includes DNL, lipid uptake and accumulation, fatty acid oxidation (FAO), and ferroptosis [59], which could be targeted (Figure 2).

### 4.1. De Novo Lipogenesis 

The reported higher lipid content of glioma cells over other intracranial tumors [53] is partly attributed to an elevated level of DNL, and also associated with the accumulation of FAs. Excess FAs can either be stored in the form of triacylglycerols (TAGs) in lipid droplets (LDs), be oxidized to provide energy, or be involved in several cellular activities via signaling [60,61]. DNL engages multiple enzymes including ATP-dependent citrate lyase (ACLY), acetyl-CoA carboxylase (ACC), and fatty acid synthase (FASN) [62]. ACLY is a cytoplasmic enzyme responsible for starting DNL by producing acetyl-CoA from citrate transported from mitochondria [63]. ACC then irreversibly carboxylates acetyl-CoA into malonyl-CoA [64], followed by a FASN-mediated condensation reaction between acetyl-CoA and malonyl-CoA to yield fatty acids up to palmitate [65]. An increased ACLY activity within the pseudopodia of GBM cells has been observed. Moreover, analysis of the gene expression database confirmed a correlation between ACLY expression and poor prognosis. In accordance, inhibition of ACLY by hydroxycitrate caused reduced migration of GBM cells *in vitro.* Therefore, ACLY was suggested as a potential target to suppress hypoxic cell invasion [66]. Similarly, inhibition of ACC by siRNA or by ACC inhibitor led to the impaired proliferation and viability of U87 EGFRvIII glioblastoma cells by hampering DNL, reducing cellular respiratory control ratio, and causing membrane permeability loss [67]. FASN has been found to be upregulated in both glioblastoma cell lines and human glioma tissues compared with normal rat astrocytes and normal human brain, respectively. Similarly, inhibition of FASN by cerulenin caused a 50% reduction in DNL and a decrease in cell viability [68]. Treating glioma stem cells with cerulenin resulted in the inhibition of migration and proliferation of the cells [69]. In a clinical study on patients of first-relapse GBM, co-administration of the FASN inhibitor TVB-2640 and bevacizumab significantly improved the progression-free survival at 6 months compared with bevacizumab treatment alone [70]. A further clinical trial is ongoing to investigate the effect of the oral, selective small-molecule inhibitor of FASN, ASC40 and bevacizumab (Table 1). Altogether, these findings suggest that targeting DNL may be a promising approach to reduce GBM aggressiveness.

Lipogenesis and cholesterol homeostasis are regulated by a family of transcription factors, called sterol regulatory element-binding proteins (SREBPs). SREBPs are synthesized as precursors complexed with the SREBP cleavage activating protein in the endoplasmic reticulum (ER) membrane. These transcription factors need to be transported from the ER to the Golgi apparatus and cleaved by site-1 and -2 proteases (S1P/S2P) [71]. SREBP-1 specifically activates the responsible genes of the enzymes involved in the DNL, whereas SREPB-2 is responsible for cholesterol homeostasis [72]. SREBPs represent an attractive pathway to target in the treatment of GBM as several studies have concluded that SREBP-1 is highly active in GBM cells [73]. Accordingly, apoptosis and decreased viability of GBM cells were observed upon inhibition of SREBP activation by using PF-429242, an S1P inhibitor [74].

### 4.2. Lipid Uptake and Storage

Generally, tumor cells do not rely only on the DNL process to supply the required lipids but also on lipid uptake from the extracellular milieu [75]. Several studies showed important roles for CD36, fatty acid binding proteins (FABPs), and low-density lipoprotein (LDL) receptors in GBM. CD36 is a scavenger receptor responsible for, among other functions, binding and transportation of long-chain free fatty acids. CD36 is expressed on the surface of several cells including microglia [76]. CD36 has also been shown to be highly expressed on the surface of GBM stem cells. Moreover, its knockdown resulted in the inhibition of self-renewal and tumor initiation abilities of GBM cells. Based on clinical datasets and experimental data expression levels of CD36 in GBM, it has been proposed as a prognostic marker for patient survival [77]. FABPs are responsible for the intracellular transportation of lipids into different cellular compartments, such as LDs and ER [78]. Interestingly, FABPs are highly expressed in the glial cells of the developing brain; however, it is barely detectable after maturation [78]. In comparison with normal cells, the expressions of both FABP4 and FABP5 have been found to be higher in GBM cells. The expression of FABP4 has been associated with a poor prognosis, while its inhibition suppressed metastases *in vivo* and *in vitro,* highlighting FABP4 as a possible target for the treatment of GBM [79,80]. Gene expression analysis of surgical specimens found an elevated expression of FABP7 in GBM samples. In addition, *in vitro* experiments showed an increased ability of patient-derived GBM cells to migrate upon overexpression of FABP7. Furthermore, its nuclear accumulation has been suggested as predictive of poor prognosis [81,82]. 

As previously mentioned, the brain produces cholesterol by DNL through the mevalonate pathway. The process begins with β-hydroxyl-β-methylglutaryl-coenzyme A (HMG-CoA) synthesis by the condensation of acetyl-CoA and acetoacetyl-CoA. Mevalonate is then formed by the action of HMG-CoA reductase (HMGCR), the rate-limiting enzyme of cholesterol synthesis, and the reaction continues to yield cholesterol [83]. Unesterified cholesterol is considered the prominent form of cholesterol in the brain, while the esterified, storage form of cholesterol accounts for only 1% of the total cholesterol content in LDs [41]. Esterification is performed by acyl-coenzyme A: cholesterol acyltransferase (also named sterol O-acyltransferase (SOAT)), which is the key enzyme of the cholesterol storage process [84] that can prevent cholesterol overload. Migration and proliferation of U251 and U87 glioblastoma cell lines were suppressed by applying simvastatin, an inhibitor of HMGCR, leading to apoptosis [85]. Another study recognized elevated SOAT expression in GBM and its inhibition blocked cholesterol esterification. Subsequently, DNL suppression was provoked through the feedback inhibition of SREBP-1, which resulted in GBM growth arrest [73].

LDs are organelles responsible for storing FAs in the form of neutral lipids, such as TAGs and cholesteryl esters, thereby protecting cells from the toxic effects of free FAs [86]. Neutral lipids form the hydrophobic core that is enveloped by phospholipids and surface proteins, such as perilipins [86]. These stored lipids could be used as substrates to build membranes or to generate energy upon nutrient shortage. To achieve this, enzymes like adipose triglyceride lipase, diacylglycerol lipase, and monoacylglycerol lipase are activated [87,88]. Formation of LDs involves several enzymes such as the abovementioned SOAT and acyl-CoA: diacylglycerol acyltransferases (DGAT 1/2), which stimulate the final process of TAG formation by adding activated fatty acids to diacylglycerol [89]. In addition, stearoyl-CoA desaturase (SCD) contributes to LD formation by stimulating the generation of MUFAs by converting saturated FAs into unsaturated FAs in the ER. In this context, stearoyl-CoA is considered the preferred substrate and is converted to oleoyl-CoA [90]. These MUFAs can also be used for the synthesis of phospholipids.

LDs are not recognized in healthy brain tissues but are highly expressed in the brain tissues of GBM patients. Accordingly, administration of oleic acid led to the accumulation of LDs in the U138 glioblastoma cell line and triggered high rates of FAO and cell migration. Furthermore, the selective inhibition of monoacylglycerol lipase by JZL184 has been shown to suppress GBM proliferation, pointing out a prominent role of reprogrammed lipid metabolism in glioma progression [91]. In addition to the key role of hypoxia in invasion and therapy resistance of GBM [92,93], lipid homeostasis is also affected by the hypoxic conditions in growing glioblastoma. It has been shown that necrotic areas contain more LDs than non-necrotic areas in GBM samples [94,95]. Peroxisome proliferator-activated receptor alpha, a transcription factor that regulates several lipid metabolizing enzymes, was found to be highly upregulated in hypoxia-treated primary GBM cells of high-grade glioma patients. Moreover, hypoxic cells showed elevated LD formation and higher levels of TAGs, cholesteryl esters, and cholesterol *in vitro* [95]. Similarly, U87-MG cell line supplementation with LDL under hypoxic conditions led to the accumulation of LDs. Additionally, patient-derived samples supplemented with LDL, incubated under hypoxic conditions, and grafted into mice brains showed LDs settling in the hypoxic niche [96]. 

GBM cells have been shown to overexpress DGAT1 to prevent lipotoxicity-induced cell death by storing excess FAs within LDs. DGAT1 inhibition led to an impairment of LD formation and caused cell death in an *in vivo* study as a result of the accumulation of reactive oxygen species [97]. Metabolic profiling of TMZ-resistant GBM cells revealed SCD-1 overexpression elsewhere. SCD-1 knockdown was shown to re-sensitize cells to TMZ treatment, while a combined therapy of TMZ and SCD-1 inhibitor was found to reduce the mobility and viability of GBM cells [98]. A study of the phospholipidome proposed that silencing SCD-1 might help to overcome GBM treatment resistance in combined therapies [99]. Moreover, several intracranial tumors including GBM were shown to display high or increased activity of the LDL receptor, a receptor responsible for cholesterol transportation, making these cells highly dependent on cholesterol supply [100]. Several attempts have been made to target the cholesterol metabolism of GBM cells, such as using a Liver X receptor agonist, which led to cell death and tumor regression *in vivo* in a cholesterol-dependent manner [101,102]. 

### 4.3. Fatty Acid Oxidation

FAO is the process by which cells produce energy using FAs as substrates. Carnitine palmitoyl transferase 1 (CPT1) is one of the enzymes involved in FAO, converting long-chain acyl-CoA to acylcarnitine for mitochondrial transport. Glioblastoma specimens were found to express higher and lower expression levels of the isoforms CPT1A and CPT1C compared with low-grade gliomas, respectively [103]. Medium-chain acyl-CoA dehydrogenase (MCAD) is another enzyme involved in the FAO process. MCAD is upregulated in GBM compared with normal brain tissues. Depletion of MCAD caused the accumulation of medium-chain fatty acids and subsequent cell death of GBM cells. The authors suggested that in tumor types surrounded by a lipid-rich environment, such as GBM, targeting MCAD may be an effective approach to hinder tumor progression [104]. In fact, inhibition of FAO by etomoxir reduced cell viability and proliferation in a syngeneic mouse model and led to ATP depletion and subsequent cell death in human GBM cells *in vitro* [105,106]. 

### 4.4. Ferroptosis

Ferroptosis is a special form of cell death characterized as a non-apoptotic, iron-dependent process that causes lipid peroxidation-mediated cell death. The three main components of ferroptosis are iron, PUFAs, and glutathione peroxidase (GPX) [107]. The process starts with the Fenton reaction, where intracellular iron reacts with hydrogen peroxide yielding hydroxyl radicals (•OH). •OH radicals react with PUFAs, resulting in phospholipid hydroperoxides (PLOOH) that are normally removed and converted to PLOH by scavengers such as GPX4. Unscavenged PLOOH, however, can react with iron to form radicals like alkoxyl (PLO•) and peroxyl (PLOO•), which then further react with PUFAs. Repeated reaction cycles lead to the accumulation of peroxidized lipids that finally cause membrane disruption and cell death [108]. As a newly discovered type of cell death, ferroptosis has been proposed as an attractive target in GBM [109]. In support of this, ferroptosis-related genes have recently been indicated with a prognostic value for GBM [110]. Moreover, the SERBP inhibitor fatostatin has been shown to trigger ferroptosis in GBM cell lines by hindering GPX4 synthesis [111].

## 5. Lipids with Special Impact on Glioblastoma Progression

### 5.1. Prostaglandins 

Prostaglandins (PGs) are AA-derived inflammatory mediator lipids that have a primary role in regulating biological activities in both healthy and inflammatory conditions. Their synthesis starts with the action of PLA_2_ releasing AA from the *sn-2* position of phospholipids. Cyclooxygenase isoenzymes (COX-1 and COX-2) then metabolize AA into different types of PGs [112]. It has been raised that high expression of COX-2 in GBM tissues enhances migration through prostaglandin E2 (PGE_2_) [113]. Furthermore, a strong correlation has also been observed between COX-2 expression and poor survival [114]. Therefore, there have been several attempts to target PG synthesis and action in GBM. A higher expression level of the membrane-associated prostaglandin E synthase 1 (mPGES-1) has been noticed in the U87-MG cell line compared with primary astrocytes. mPGES-1 knockdown reduced cell growth and proliferation by inhibiting the activation of protein kinase A. Externally administrated PGE_2_ retrieved cellular growth and proliferation [115]. By comparing metabolome and gene expression profiles of chemotherapy-sensitive vs. resistant GBM cells, PGE_2_ has been shown to participate in the development of TMZ resistance via the COX-2 pathway [116]. In contrast, it was shown elsewhere that primary cultures of GBM tumors with higher levels of mPGES-1 were more prone to Bax-dependent apoptosis [117]. Furthermore, AA treatment was shown to inhibit the proliferation of GBM cell lines LN229 and HNGC2 in an *in vitro* study. Nevertheless, the authors suggested that AA affects cell proliferation independent of its metabolites [118]. Altogether, PGE2 can exert both pro- and antitumor effects depending on the cellular or molecular context. 

### 5.2. Lysophosphatidic Acid

GBM cells can also increase their motility and metastatic potential through lysophosphatidic acid (LPA) and its receptors [119]. These effects could be attributed to an increased expression level of autotaxin (ATX), the phosphodiesterase enzyme that converts lipid substrates such as lysophosphatidylcholine to LPA. Accordingly, genetic downregulation of ATX blocked GBM cell migration *in vitro* [120]. In addition, PF-8380, an ATX inhibitor, improved sensitivity toward radiotherapy and decreased GBM invasion in cell line and mouse models [121]. 

### 5.3. Endocannabinoid Lipids

Beyond an important neuromodulatory role, it has been shown that ECs have antitumor effects against various cancer types [122]. Mostly AEA and to some extent 2-AG have been investigated for their antitumor role. Nevertheless, Δ9-tetrahydrocannabinol (THC) and cannabidiol (CBD), phytocannabinoids derived from *Cannabis sativa*, have been clinically tested for GBM treatment for both their palliative and antitumor effects. In a clinical study with a cohort of GBM patients, THC was safely administered locally with beneficial effects on suppressing tumor growth [123,124]. Released in a sustained manner, THC-loaded microparticles were actively delivered to the tumor site in a murine GBM model, where they reduced viability [125]. Furthermore, THC also synergized and improved the antitumor activity of TMZ, when applied in combination [126]. In line with these findings, intracranial application of THC was shown to improve the apoptotic activity of cleaved caspase 3 [127]. The mechanisms by which THC provokes cell death involve autolysosome permeabilization, cathepsin release [128], and subsequent ER stress-dependent autophagy [129]. Oral administration of CBD in addition to the standard treatment has been shown to extend patient survival [130]. To reduce the psychoactive effect of THC, a combination of THC and CBD has been clinically tested in the form of a nabiximols spray added to TMZ for combating recurrent GBM. The results of a placebo-controlled phase I trial showed that nabiximols was tolerable and improved overall survival. However, further studies with a higher number of patients should be performed to confirm these results [131].

**Figure 3:**
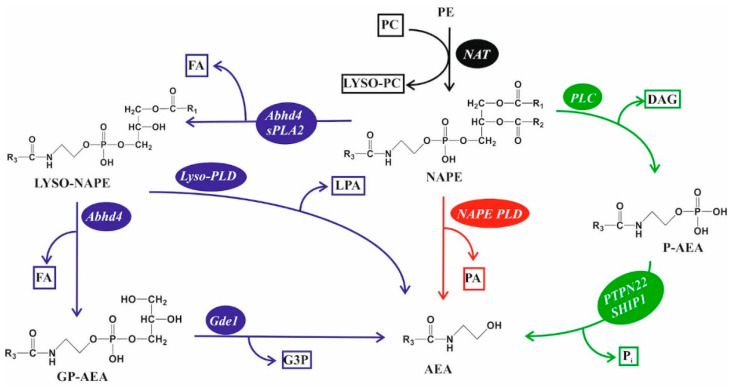
Alternative NAPE metabolic routes to AEA. (Black) N-arachidonoyl-ethanolamine (NAPE) is made from phosphatidylethanolamine (PE) by N acylation of the PE head group, which is mediated by N-acyl transferases (NATs) (e.g., PLA2G2E, PLA2G4E) using phosphatidylcholine (PC) as a fatty acid source. (Red) NAPE is directly metabolized to anandamide (AEA) and phosphatidic acid (PA) by phospholipase D (NAPE-PLD) [132]. (Green) Phospholipase C (PLC) activities can produce phospho-AEA (P-AEA), which is further metabolized to anandamide (AEA) and inorganic phosphate (P_i_) by protein tyrosine phosphatase non-receptor type 22 (PTPN22) and SH2 domain-containing inositol 5′ phosphatase-1 (SHIP1) [133,134]. (Blue) Fatty acids of NAPE can be cleaved by phospholipase A2 (PLA_2_) activities of abhydrolase domain-containing 4 (Abhd4) or secretory phospholipase A2 (sPLA_2_), yielding lyso-NAPE. Lyso-NAPE may be further metabolized to AEA directly with lysophosphatidic acid (LPA) as a side product. Lyso-NAPE may also be metabolized to glycerophospho-AEA (GP-AEA) and fatty acid (FA) by Abhd4, and then further to AEA and glycerol-3-phosphate (G3P) by glycerophosphodiester phosphodiesterase 1 (Gde1) [134,135].

AEA has been a focus of physiological and tumor brain research. To our current understanding, AEA is synthesized from N-arachidonoyl-ethanolamine (NAPE) via multiple, direct, and indirect pathways (Figure 3). Although the role of these alternative pathways is not understood, degradation of NAPE yields several metabolites, some of which are potent signaling molecules, such as DAG, LPA, and AEA. Interestingly, several NAPE metabolizing enzymes have been implicated as potential tumor suppressors as well [132,133,134,135], but the potential role of NAPE metabolites in tumor progression is completely unknown. Similar to its synthesis, AEA is degraded by multiple pathways. These include its hydrolyzation to AA and ethanolamine by fatty-acid amide hydrolase (FAAH) [136] or by N-acylethanolamine-hydrolyzing acid amidase (NAAA) [137], or by its oxygenation via lipoxygenases that yield hydroperoxyl compounds [138]. In addition, COX-2 can metabolize AEA to prostanoids, mainly to PGE2 ethanolamide [139]. An up to 17-fold increase in NAPE and AEA levels has been shown in the tumor tissue of GBM patients compared with normal brain lipid concentrations, which was accompanied by a decrease in the efficacy of AEA-degrading enzymes [140]. In a later study, decreased levels of AEA were reported in GBM samples, but the authors noticed significant differences in sample collection, handling, and measurement protocols of the sensitive samples [141]. 

Little is known about the mechanism of AEA-mediated effects on brain tumors, in particular GBM. An early report on the antitumor effect of AEA has shown inhibition of MCF-7 and EFM-19 breast cancer cells via a cannabinoid receptor-1 (CB1)-dependent mechanism [142]. Later, AEA-induced apoptosis in neuroblastoma and lymphoma cell lines was shown to be abolished by the antagonism of vanilloid receptors [143]. C6 glioma proliferation was restricted *in vitro* by exogenously administered AEA in a cannabinoid and vanilloid receptor-dependent manner. It was also shown that a metabolically stable analog meAEA (R-(+)-methanandamide) had much lower efficacy [144]. meAEA induced COX-2 expression in human neuroglioma cells even upon treatment with a selective CB1 receptor antagonist, suggesting a cannabinoid receptor-independent mechanism [145]. Other enzymes were found to contribute to AEA activities because inhibition of COX-2 and lipoxygenase reduced the cytotoxic effect of AEA on A375 melanoma cells, whereas FAAH antagonism had the opposite effect [146]. Similar results were achieved when the AEA analog Met-F-AEA (2-methyl-2′-F-anandamide) was combined with an FAAH blocker (URB597), causing a regression of non-small-cell lung cancer cells [147]. In addition, oxidative stress has also been implicated in AEA-mediated antitumor effects, as antioxidants mitigated oxidative stress-mediated apoptosis triggered by AEA in non-melanoma skin cancer even with the blockage of cannabinoid and vanilloid receptors, suggesting a yet unraveled antitumor mechanism of AEA [148]. 

## 6. Clinical Tests of Lipid-Targeting Drugs for Glioblastoma Treatment

As described above, several lipid-based targets have been raised in *in vitro* and preclinical studies for combating GBM. To evaluate the therapeutic potential of these lipid targets, a small number of clinical trials have been conducted so far (Table 1). Lipid metabolism-related drugs have been used in combination with conventional radio- and chemotherapy. The FASN inhibitor TVB-2640 was shown to improve progression-free survival measured at 6 months in a phase II study [70], while the effect of another FASN inhibitor, ASC40, is currently being tested in a phase III trial. The use of the HMGCR inhibitor atorvastatin, which was supposed to reduce DNL, has failed to confer any clinical benefit [149]. Neither inhibition of COX-2 by celecoxib, which was expected to block PG synthesis and AEA metabolism, resulted in a significant clinical benefit [150]. Early-stage studies indicate that CBD and THC could have a beneficial effect on the overall survival of GBM patients [130,131]. 

**Table 1:** Clinical trials targeting lipid metabolism in GBM. THC: Δ9-tetrahydrocannabinol, CBD: cannabidiol, FASN: fatty acid synthase, HMGCR: β-hydroxyl-β-methylglutaryl-CoA reductase, COX-2: cyclooxygenase 2, TMZ: temozolomide, PFS6: progression-free survival at 6 months, and OS: overall survival.

NCT No.	Drug	Target	Combined with	Phase	Outcome	Reference
NCT03032484	TVB-2640 (Denifanstat)	FASN	Bevacizumab	II	Improved PFS6	[70]
NCT05118776	ASC40	FASN	Bevacizumab	III	Ongoing	-
NCT02029573	Atorvastatin	HMGCR	Radiotherapy +TMZ	II	No benefit	[149]
NCT00047294	Celecoxib	COX-2	Thalidomide +TMZ	II	No benefit	[150]
Case study (15 patients)	CBD	Cannabinoid pathway	Radiotherapy +TMZ	-	Improved OS	[130]
NCT03529448	TN-TC11G (THC+CBD)		Radiotherapy +TMZ	I	Ongoing	-
NCT01812616	Nabiximols		TMZ	Ib	Improved OS	[131]

## 7. Conclusions

Aggressiveness and poor prognosis of GBM, as evidenced by short survival after diagnosis and short-term effectiveness of therapies, continue to represent a heavy burden on the medical community and patients. To overcome this health and social challenge, serious efforts have been made to treat GBM by exploiting its various characteristics. Among them, reprogramming or attacking the pathophysiological lipid metabolism appears to be an attractive novel therapeutic possibility. This review highlights much of the related research that investigated various pathways of GBM lipid metabolism. Inhibition or elimination of several enzymes or participating metabolites may counteract growth and invasion, block proliferation, or induce apoptosis of GBM cells. Altogether, GBM is embedded in a fairly lipid-rich brain environment and is highly dependent on lipid supply and exchange, therefore lipid-based therapeutic approaches are emerging and gaining more importance.
